# Migration background and use of preventive healthcare services: Findings of the German Ageing Survey

**DOI:** 10.1186/s12889-024-19927-3

**Published:** 2024-09-09

**Authors:** Arianit Kameraj, Hans-Helmut König, André Hajek

**Affiliations:** Department of Health Economics and Health Services Research, University Medical-Center Hamburg-Eppendorf, Hamburg Center for Health Economics, Hamburg, Germany

**Keywords:** Migration, Flu vaccinations, Influenza, Cancer screenings, Health check-ups

## Abstract

**Background:**

This study aimed to investigate the relationship between migration background and the utilization of preventive healthcare services.

**Methods:**

Cross-sectional data from wave 5 in the year 2014, with a sample size of 7,684 individuals, were extracted from the nationally representative German Ageing Survey. The survey included community-dwelling individuals aged 40 years and above, with migration background serving as the primary independent variable. The outcomes measured included participation in cancer screenings, flu vaccinations, and routine health check-ups. Multiple logistic regressions were used to examine the association between migration background and preventive healthcare services.

**Results:**

Regressions showed that the presence of a migration background with personal migration experience (compared with not having a migration background) was associated with a lower likelihood of using preventive healthcare services. More precisely, compared to individuals not having a migration background, individuals with a migration background and personal migration experience had a lower likelihood of routine health check-ups (OR = 0.76, 95% CI: 0.61 to 0.95), flu vaccinations (OR = 0.75, 95% CI: 0.59 to 0.95) and cancer screenings (OR = 0.71, 95% CI: 0.57 to 0.89). In contrast, there was no significant association between the presence of a migration background without personal migration experience (compared with not having a migration background) and the use of preventive healthcare services.

**Conclusion:**

In conclusion, results showed differences between individuals without migration background and individuals with migration background (and with personal migration experience) in terms of using preventive healthcare services. It may be helpful to specifically address individuals with migration background (and with migration experience) in terms of using preventive healthcare services.

**Supplementary Information:**

The online version contains supplementary material available at 10.1186/s12889-024-19927-3.

## Introduction

Preventive healthcare services, particularly encompassing flu vaccinations, cancer screenings, and check-ups, play a pivotal role in sustaining optimal health and forestalling the onset of severe medical conditions. The ‘Check-up 35’ is a preventive service comprising a comprehensive array of examinations, tests, and screenings designed for adults aged 35 and above. These include physical examinations, blood pressure measurements, blood tests (complete lipid profile covering total cholesterol, LDL and HDL cholesterol, and triglycerides), urine tests, and other health assessments tailored to the individual risk profile of the patient [1].

In Germany, statutory health insurance (Gesetzliche Krankenversicherung, or GKV) and public regulations work together to regulate preventive healthcare services. The GKV system provides coverage for a variety of preventive services to advance the health and wellbeing of those with statutory health insurance. The following are some essential components of Germany’s regulations on preventive healthcare services.

GKV is a requirement for people whose incomes are below a certain level and is a source of coverage for the majority of Germans. They offer coverage for a variety of healthcare services, including preventative measures. The German GKV system provides coverage for a range of preventive healthcare services, including but not limited to (please see the methods section for further details):

- Vaccinations: There are schedules in place for both children and adults for receiving vaccinations against common diseases.
- Regular cancer screenings: are offered, including those for breast, cervical, and colon cancers, which are common cancers.
- Health check-up: To monitor their overall health status, insured people are entitled to health examinations every three years.
- Dental Check-ups: Preventive healthcare also includes cleanings and examinations of the mouth.

The Federal Joint Committee, also known as the “Gemeinsamer Bundesausschuss”, or “G-BA”, is in charge of deciding which preventive services are covered and setting directives. To reflect developments in science and medicine, these guidelines may be updated and changed from time to time.

The statutory health insurance does cover a wide range of preventive services but depending on the person’s health insurance or insurance provider, there may be differences in the specific services covered or additional options available. Additionally, certain individuals (e.g. self-employed individuals, high-income earners, civil servants) can choose private health insurance (Private Krankenversicherung, or PKV).

Thus far, most preventive care services are infrequently used [2]. For example, in Germany, the use of flu vaccinations, cancer screening programs, and preventive check-ups in Germany is as follows: According to the Robert Koch Institute’s (RKI) GEDA study, 55.4% of respondents aged 35 and over indicated that they had undergone health check-ups at some point [3]. According to the most recent data, from 2018 to 2020, approximately 46–72% of insured individuals, eligible for the Check-Up 35 examination and aged 35 or older, participated in it within 4 years among members of the AOK (Allgemeine Ortskrankenkasse) [4]. With regard to cancer screenings, a study based on data from the Central Institute of the Association of Statutory Health Insurance Physicians reported a participation rate in the year 2015, for example, for mammography screening programs of 51.5% and a participation rate in the year of 2016 for early detection of prostate cancer of 24.2%. Men between the ages of 45 and 49 had the lowest utilization rates. Participation rates increase to over 30% for those over the age of 65 [5]. From 2009 to 2020, annual age-specific usage rates changed little over time for women and men [4]. With regard to flu vaccination, according to the RKI, approximately 50% of the population in Germany were vaccinated against the flu in 2020 [6, 7].

While the evidence for the benefits of general health check-ups in Germany is critically discussed for a variety of reasons [8], there is clear evidence for the benefits of cancer screenings, for example [9].While several determinants, e.g., being female or higher education, of the use of preventive healthcare services have been identified [10, 11], other determinants are poorly understood. For example, there has been little research on the link between immigrant background and use of preventive healthcare services (see [12]). Moreover, many of these studies have focused on specific prevention strategies [13], specific immigrant groups [14], or single sexes [15]. Based on large-scale census data, a former study showed that women migrating from EU and non-EU countries are less likely to participate in screening [16]. There are survey results on the use of preventive healthcare measures among migrants [17]. However, such research is commonly restricted in sample size. Former German studies have shown that migrants have a lower likelihood of using cancer screenings compared to individuals without migration background, especially for female migrants [18, 19].

Another study examined health care utilization among immigrants, distinguishing between first- and second-generation immigrants. The results show that first-generation immigrants have different health care utilization patterns than native Germans and second-generation immigrants, with greater reliance on general practitioners and less reliance on specialists. Ensuring equal access to care for first-generation immigrants is important [20].

A study with a few other outcomes was published in 2021, in which methodological care was taken to ensure that people with a migration background were adequately taken into account through oversampling. This study provided evidence that migrants (especially first-generation migrants with a length of stay of at least 20 years in Germany) make less use of preventive care [21]. Immigrant groups may face many barriers in accessing and using healthcare, including language barriers, cultural differences, and disparities in socio-cultural factors [22–24]. These factors can significantly influence how migration affects preventive healthcare practices and may be particularly relevant for preventive healthcare services, as they often require proactive engagement with the healthcare system and may involve additional steps such as making appointments and understanding the importance of certain screenings or vaccinations. In sum, there are very few German studies (partly with restricted sample sizes and restricted number of preventive healthcare services) examining the association between having a migration background and using preventive healthcare services. Thus, the objective of this study is to examine the association between migration background and the use of different preventive healthcare services based on a large, nationally representative sample of middle-aged and older adults in Germany.

Investigating the association between migration and use of preventive healthcare services is important because Germany has a large proportion of individuals with a migration background. Moreover, this knowledge can help identify possible inequalities in access to health care services. In a second step, this helps target specific individuals who may not be using preventive care services on a regular basis.

## Methods

### Sample

Our study follows the STROBE guidelines for the reporting of cross-sectional studies (please see the Supplementary File 1).

The cross-sectional data came from the German Ageing Survey (DEAS) in 2014. At eight points in time (1996, 2002, 2008, 2011, 2014, 2017, 2020 and 2020/2021), representative samples of community-dwelling adults aged 40 or over in Germany were drawn using national probability sampling. In total, 10,323 people were interviewed in 2014, with 6,001 (response rate: 25%) being interviewed for the first time and the remaining 4,322 (response rate: 61%) having participated in a previous round of the DEAS study. Further details on the 2014 wave of the DEAS study have been published elsewhere [25, 26]. We used data from the 2014 wave since it had the largest sample size (compared to other waves from the DEAS study).

Eligible participants provided data on various aspects in interviews based on standardized questionnaires at their homes (10,324 individuals were interviewed). Subsequently, individuals could fill out a drop-off questionnaire (8,039 individuals filled out this questionnaire in a valid way). The questionnaire was only available in German, but they were able to get assistance from the interviewers. In the analytical sample with flu vaccinations as outcome measure, n equaled 7,684 individuals due to some missing values. Thereof, 94.7% of the individuals did not have a migration background, whereas 4.6% had a migration background with their own migration experience and 0.7% have a migration background without their own migration experience. Migration is defined as follows: People with a migration background and own migration experience (i.e. immigration to Germany) and people with a migration background but without own migration experience (i.e. born and raised in Germany). The data on place of birth, year of immigration, possession of German or foreign citizenship and naturalization experience were used for this purpose.

A disproportionate random selection of address information from resident registration offices is used to create the DEAS sample. Data regarding migration history is not included in the information found in these records. As a result, the register-based sampling cannot take migration background into account [27]. It is crucial to recognize the significant efforts made to persuade people to participate, including migrants, through programs like persistent contact attempts.

The participants provided their written informed consent before participating in the study. An ethics vote was not obtained because the criteria for such a vote were not fulfilled (e.g., use of invasive methods).

### Dependent variables

In our current study, we assessed three key outcomes: the utilization of routine health check-ups, participation in regular cancer screenings, and receipt of regular flu vaccinations. Concerning health check-ups, participants were asked the question: “In the past years, did you consistently undergo regular health check-ups?” Response options included checkboxes for “yes” or “no”. Previously, the age limit for a health check-up was 35, which was also the case during the period of our survey. Since 2019, i.e. after the time of our survey, people between the ages of 18 and 34 now also can have a health check-up.

Similarly, inquiring about cancer screenings, participants were presented with a parallel question: “In the past years, did you routinely engage in early cancer screenings?” Response options allowed participants to indicate either “yes” or “no”. Most cancer screenings are recommended from the age of 50.

Likewise, in the context of flu vaccinations, participants were also asked: “In the past years, did you consistently receive regular flu vaccinations?” Participants had the option to select either “yes” or “no” from the provided checkboxes. This involves regular flu vaccinations in accordance with the STIKO recommendations.

### Independent variable of interest: Migration background

We categorized participants into three distinct groups:

1. Individuals without a migration background.
2. Individuals with a migration background (and with personal migration experience): In short: Individuals with personal migration background.
3. Individuals with a migration background (and without a personal migration experience): In short: Individuals with non-personal migration background.

As per the DEAS guidelines, it should be noted that immigration prior to 1950 does not count as migration background. Additionally, individuals born in the former eastern regions (or the “German Reich”) and those immigrating to either the GDR (German Democratic Republic) or FRG (Federal Republic of Germany) after the year 1949 are considered individuals with a migration background.

### Covariates

Following prior research in this area [25], we adjusted for the following sociodemographic and health-related factors in regression analysis: age in years, gender (male, female), family status (married and living with spouse, married and living apart from spouse, divorced, widowed, single), and level of education by ISCED (Internationally Standard Classification of Education), 1 (low = ISCED 0–2) respondents without completed vocational qualification and without A-levels (Abitur); 2 (medium = ISCED 3–4) respondents with vocational qualifications or respondents with A-levels (Abitur) but no vocational qualifications or degrees; 3 (high = ISCED 5–6) respondents with university degrees or master craftsmen. Moreover, we adjusted for self-rated health (single item, from 1 = very good to 5 = very bad) and the number of chronic conditions (count score ranging from 0 to 11, including the following chronic conditions: Cardiac and circulatory disorders; bad circulation; joint, bone, spinal or back problems; respiratory problems, asthma, shortness of breath; stomach and intestinal problems; cancer; diabetes; Gall bladder, liver or kidney problems; bladder problems; eye problems, vision impairment; ear problems, hearing problems).

### Statistical analysis

First, sample characteristics are shown - also stratified by migration background. Thereafter, multiple logistic regressions were performed to examine the link between migration background and preventive healthcare services (i.e., routine check-ups, flu vaccinations and cancer screenings). In regression analysis, we adjusted for age, sex, education, marital status, self-rated health, and the number of chronic conditions. Listwise deletion was used to handle missings since the proportion of missings was very low. Some sensitivity analyses were conducted (please see Sect. Multiple regression analysis for further details). The aim of these sensitivity analyses was to check the robustness of our results (when restricted to certain age groups). Of note, sensitivity analyses (e.g., age restrictions) were not conducted when routine health check-ups served as outcome because these check-ups are recommended for individuals aged 35 years and over – and the DEAS study focused on individuals aged 40 years. Therefore, it is recommended for all individuals included in the DEAS study.

The level of statistical significance was determined at *p* < 0.05. All statistical analyses were performed using Stata 16.1 (Stata Corp., College Station, Texas).

## Results

### Sample characteristics

Table 1 shows the sample characteristics stratified by migration background (*n* = 7,684 individuals). The total sample was aged from 40 to 85 with a mean age of 64.4 (SD: 11.2) years. A slight minority of about 49% of all participants was male and about 70% of the individuals were married and living in a household together. Regarding educational attainment, 51.5% of the individuals were moderately educated (ISCED 3–5) and 42% had a high level of education (ISCED 5–6), with significant differences within the groups. No significant differences were found in terms of self-rated health and the number of chronic conditions.

**Table 1:** Sample characteristics (also stratified by migration background)

Columns by: migration background (MB)	0. No MB	1. MB with experience	2. MB without experience	Total	*P*-value
n (%)	7,276 (94.7)	357 (4.6)	51 (0.7)	7,684 (100.0)	
Sex, n (%)					0.04
1. Men	3,600 (49.5)	152 (42.6)	24 (47.1)	3,776 (49.1)	
2. Women	3,676 (50.5)	205 (57.4)	27 (52.9)	3,908 (50.9)	
Age, mean (sd)	64.6 (11.2)	60.4 (11.0)	63.2 (13.5)	64.4 (11.2)	< 0.001
Family status, n (%)					0.38
1. married and living with spouse	5,093 (70.0)	247 (69.2)	36 (70.6)	5,376 (70.0)	
2. married and living apart from spouse	115 (1.6)	7 (2.0)	1 (2.0)	123 (1.6)	
3. divorced	725 (10.0)	44 (12.3)	9 (17.6)	778 (10.1)	
4. widowed	821 (11.3)	38 (10.6)	2 (3.9)	861 (11.2)	
5. single	522 (7.2)	21 (5.9)	3 (5.9)	546 (7.1)	
Education, n (%)					< 0.001
1. low, ISCED 0–2	417 (5.7)	75 (21.0)	4 (7.8)	496 (6.5)	
2. medium, ISCED 3–4	3,766 (51.8)	166 (46.5)	28 (54.9)	3,960 (51.5)	
3. high, ISCED 5–6	3,093 (42.5)	116 (32.5)	19 (37.3)	3,228 (42.0)	
Self-rated health (from 1 = very good to 5 = very bad), mean (sd)	2.5 (0.8)	2.5 (0.8)	2.6 (1.0)	2.5 (0.8)	0.77
Number of chronic conditions, mean (sd)	2.6 (1.9)	2.5 (2.0)	2.8 (1.9)	2.6 (1.9)	0.46
Regular cancer screenings, n (%)					< 0.01
No	2,464 (34.4)	149 (42.5)	13 (27.7)	2,626 (34.8)	
Yes	4,691 (65.6)	202 (57.5)	34 (72.3)	4,927 (65.2)	
Regular flu vaccinations, n (%)					< 0.001
No	4,053 (55.7)	243 (68.1)	29 (56.9)	4,325 (56.3)	
Yes	3,223 (44.3)	114 (31.9)	22 (43.1)	3,359 (43.7)	
Regular health check-ups, n (%)					< 0.01
No	2,483 (34.5)	151 (43.1)	17 (34.7)	2,651 (34.9)	
Yes	4,708 (65.5)	199 (56.9)	32 (65.3)	4,939 (65.1)	

The total participation in the target outcomes is distributed as follows: 65.2% of the individuals had regular cancer screenings, 43.7% of the individuals received regular flu vaccinations and 65.1% of the individuals had regular routine health check-ups.

Stratified by migration background: 57.5% of the individuals with personal migration experience had regular cancer screenings, 72.3% of the individuals with non-personal migration background had regular cancer screenings and 65.6% of the individuals without migration background had regular cancer screenings. In sum, 31.9% of the individuals with personal migration experience had regular flu vaccinations, 43.1% of the individuals with non-personal migration background had regular flu vaccinations and 44.3% of the individuals without migration background had regular flu vaccinations.

Additionally, 56.9% of the individuals with personal migration experience had regular routine health check-ups, 65.3% of the Individuals with non-personal migration background had regular routine health check-ups and 65.5% of the individuals without migration background had regular routine health check-ups.

Significant differences in use of preventive healthcare services were found depending on the migration background, with a p-value of < 0.01 for regular cancer screenings, *p* < 0.001 or regular flu vaccinations, and *p* < 0.01 for regular check-ups.

Further details are provided in Table 1.

### Multiple regression analysis

Table 2 shows the results of a multiple logistic regression analysis with the regular use of preventive healthcare examination (routine check-ups, flu vaccinations and cancer screenings) as dependent variables and the migrations background as the independent variable. In all regressions, we adjusted for age, sex, education, marital status, self-rated health, and the number of chronic conditions.

**Table 2:** Association between migration background and preventive healthcare services. Results of multiple logistic regression analysis

Independent variables	Regular health check-ups	Regular flu vaccinations	Regular cancer screenings
Migration: With migration background and personal migration experience (Reference category: Without migration background)	0.76*	0.75*	0.71**
	(0.61–0.95)	(0.59–0.95)	(0.57–0.89)
- With migration background and no personal migration experience	1.01	0.99	1.32
	(0.56–1.82)	(0.54–1.82)	(0.68–2.54)
Covariates	✓	✓	✓
Constant	0.75	0.01***	0.54**
	(0.16)	(0.00)	(0.12)
Observations	7,648	7,684	7,610
Pseudo R²	0.01	0.09	0.04

Adjusted for: Age, sex, education, marital status, self-rated health, and the number of chronic conditions; Odds ratios are reported; 95% CI in parentheses; *** *p* < 0.001, ** *p* < 0.01, * *p* < 0.05, + *p* < 0.10

Regression analysis revealed a significant association between the presence of a migration background with personal migration experience (compared with not having a migration background) and a lower likelihood use of preventive healthcare services. More precisely, the association was OR = 0.76 (95% CI: 0.61 to 0.95) with routine health check-ups as outcome measure. Furthermore, it was OR = 0.75 (95% CI: 0.59 to 0.95) with flu vaccinations as outcome measure. Lastly, it was OR = 0.71 (95% CI: 0.57 to 0.89) with cancer screenings as outcome measure. No significant differences were found between the group of non-migrants and migrants without own migration experience.

As seen in Table 3 in sensitivity analysis, we followed the STIKO recommendations regarding flu vaccinations. Thus, we restricted our sample to individuals aged 60 years and over (when flu vaccinations served as outcome measure). In this regression analysis, no significant difference could be observed between migrants with their own migration experience and individuals without a migration background (OR: 0.74, 95% CI: 0.54 to 1.00) regarding flu vaccinations. Furthermore, we supplement our analysis for cancer screenings from the age of 50. From then on, the most frequently performed screenings, such as breast cancer and colorectal cancer screenings, are usually recommended. However, these may vary depending on individual risk factors. In this regression analysis, a significant difference was identified between migrants with their own migration experience and individuals without a migration background (OR: 0.66, 95% CI: 0.52 to 0.85) regarding cancer screenings.

**Table 3:** Association between migration background and preventive healthcare services. Results of multiple logistic regression analysis

Independent variables	Regular flu vaccinations (only among individuals aged 60 years and over)	Regular cancer screenings (only among individuals aged 50 years and over)
Migration: With migration background and personal migration experience (Reference category: Without migration background)	0.74+	0.66**
	(0.54–1.00)	(0.52–0.85)
- With migration background and no personal migration experience	1.10	1.17
	(0.51–2.38)	(0.56–2.41)
Covariates	✓	✓
Observations	4,981	6,773
Pseudo R²	0.05	0.04

Adjusted for: Age, sex, education, marital status, self-rated health, and the number of chronic conditions; Odds ratios are reported; 95% CI in parentheses; *** *p* < 0.001, ** *p* < 0.01, * *p* < 0.05, + *p* < 0.10

Additional File 1. STROBE Guidelines for cross-sectional studies

## Discussion

Drawing upon a comprehensive representative sample, our study aimed to explore the association between migration background and the utilization of preventive healthcare services. Our regression analyses revealed that individuals with a migration background and personal migration experience, when compared to those without a migration background, exhibited a diminished likelihood of engaging in preventive healthcare services. Conversely, no statistically significant distinctions were observed in the utilization of preventive healthcare services between individuals without a migration background and those with a migration background but without personal migration experience. One plausible explanation for the differences in terms of use of preventive healthcare services by people with migration background and personal migration experience and individuals without a migration background is health literacy. Health literacy refers to the ability to access, understand and use of information to promote and maintain good health. Former studies showed differences in health literacy between migrants and individuals without a migration background [28]. Previous studies also showed the importance of health literacy for use of preventive healthcare services [29, 30].

Even though individuals took part in the DEAS survey, a further plausible explanation may be that some language barriers were present. Language barriers can affect immigrant preventive health care use in many ways. First, language problems can make it difficult to understand medical information and instructions, leading to misunderstandings and lack of knowledge [31]. Second, language barriers can make migrants feel uncomfortable and unable to talk openly and honestly with health care professionals, hinder strong doctor-patient relationships, and reduce access to preventive health services [32]. Overall, the language barrier is most likely an important issue affecting the use of preventive healthcare services of immigrants [33].

Cultural values ​​and beliefs may also reduce migrants’ participation in preventive health measures, such as regular examinations and screenings. In some cultures, preventive health measures may be deemphasized or viewed as unnecessary, especially in the absence of obvious symptoms or health problems. This can lead to reluctance to seek preventive care, even when it is available and accessible [34].

Studies from neighboring countries in Europe provided similar results, often considering only individual prevention measures. For example, a paper-based survey from Ireland showed that migrants from Poland were less likely to use health services, including cancer screenings, than non-migrants [35]. A different study looked at health surveys about migrant populations’ use of preventive healthcare in Belgium, Italy, Malta, Portugal, and Spain. The results of a previous study [36] aligned with results from comparable studies. A study from Norway also examined the participation rate of breast cancer screenings among migrants and non-migrants. Women with an immigrant background were less likely to participate in breast cancer screenings [37].

In addition to health literacy, language and cultural barriers, a general lack of knowledge about the healthcare system and available preventive healthcare services can also contribute to the lower use of these services by migrants [30]. This may be due to differences in health care systems between countries, uncertainty about eligibility for services, or simply a lack of understanding of the importance of preventive care.

A survey of health status and health care utilization among German citizens and immigrants found that, despite similar overall health status, female Germans used more health facilities and prevention programs (e.g., cancer screenings or check-ups) than female immigrants. Interestingly, migration status itself was not significantly associated with physical health problems [19]. A further study examined the relationship between migration context and health status [18]. Results showed gender-specific patterns, with first-generation immigrant women having poorer overall health, lower levels of physical activity, lower alcohol consumption, limited knowledge of cancer screening programs (e.g., cancer screenings or check-ups), and lower rates of use of preventive services than non-immigrant women. Moreover, this former study showed a non-significant association between second-generation immigrant status (compared to non-immigrants) and regular use of cancer screenings in both women and men. This previous study attributes the non-significant results primarily to the lack of the following distinctions. Firstly, improved acculturation across generations is postulated, accompanied by the overcoming of access barriers as an additional factor.

Our results are particularly relevant within the current research landscape. A further indication of the complex nature of the healthcare disparities experienced by people with migration backgrounds is the observed lower likelihood of using preventive healthcare services among migrants with personal migration experience as compared to those without it. This is consistent with earlier research (e.g. [28–30]) that have shown how language hurdles and health literacy affect migrant patients’ use of preventive healthcare.

Our study sheds new light on this topic and confirms the results of previous studies. On this basis, future research could examine these determinants in more depth to better understand their complex interactions and implications for preventive health care in people with immigrant backgrounds. The absence of significant differences in the utilization of preventive healthcare services between individuals without a migration background and those with a non-personal migration background is somewhat perplexing. While we have previously discussed potential factors such as health literacy, language barriers, cultural values, and knowledge gaps, it is essential to note that the subgroup of migrants without personal migration experience, comprising only *n* = 51, is relatively small. Therefore, further investigations into this subgroup are imperative. The inconclusive findings in this subgroup suggest a need for more in-depth exploration. It is plausible that future studies, with larger sample sizes, could unveil potential shifts in preventive healthcare use among migrants without personal migration experience. This highlights the importance of ongoing research to elucidate patterns and trends that may emerge across generations within the migrant population. Moreover, guidelines and concepts for migration-sensitive health monitoring in Germany have now been developed [38].

Some strengths and limitations are worth noting. The present study examines the association between migration background and the utilization of preventive healthcare services in Germany. A strength of the study is that it is based on nationally representative data, which allows us to apply our findings to community-dwelling individuals aged 40 years and over and living in Germany. Furthermore, the study examines a range of regular used preventive healthcare services: flu vaccinations, cancer screenings, and check-ups, which allows for a comprehensive understanding of the topic. Additionally, the study considers important covariates.

It is important to acknowledge some limitations of the study. A possible limitation is that the data collected were based on self-reporting. This means that participants may have recalled their experiences inaccurately or reported things they thought were socially desirable. Therefore, participation rates for preventive measures may be slightly overestimated. It should also be noted that there is a limitation in its representation of individuals with a migration background. The underrepresentation observed in the study, possibly due to linguistic barriers and other associated factors, could have introduced challenges. Given that only those who can understand German were included in the study, we most likely even underestimated the true effects [39]. Future research should make an effort to be more inclusive (e.g., translating the questionnaire to other languages) in order to better capture the diversity of the population, including people with migrant backgrounds. Another limitation is that the study likely only attracted migrants who were more aware of the structure of the country and who were not isolated from the rest of society. This could lead to sample bias, as those who were largely isolated, such as those in parallel societies, might not have participated in the study. Compared to data from the German microcensus, the data from the DEAS study has a smaller proportion of individuals with a migration background (particularly older first-generation migrants). The cross-sectional nature of the analysis precludes causal interpretations of our findings. Longitudinal studies are needed in the future to better understand the relationship between variables over time.

It should be remembered that individuals may have slightly different interpretations of the term “regularly used” and that personal health conditions, beliefs and medical advice may affect an individual’s interpretation of the frequency of use of health care services.

In addition, the diversity of the migrant population resulting from their diverse backgrounds and places of origin should be taken into account. Each migrant brings with them distinct cultural, socioeconomic, and healthcare-related influences from their various countries of origin [40]. Our complete knowledge of how migration-related factors affect healthcare utilization may be restricted if this diversity is not taken into consideration. It is important to recognize that, within these groups, there can be differences that deserve attention.

Overall, the present study can contribute to our existing knowledge in this field, however, it is important to consider the strengths and limitations outlined above when interpreting the results.

## Conclusion and future research

Our results showed discrepancies between individuals without migration background and individuals with migration background (and with personal migration experience) in terms of using preventive healthcare services.

It may be helpful to understand and address cultural factors that influence the use of preventive healthcare services - specifically regarding individuals with migration background (and with migration experience) in terms of using preventive healthcare services. This may include working with culturally competent healthcare providers, working with community leaders/members and social media influencers to promote the benefits of preventive care, and addressing cultural misconceptions and beliefs that may inhibit participation in preventive care [34, 41]. Another possibility would be to target cultural centers and other gathering places through the distribution of flyers, lectures, or similar means, to raise awareness of the offerings.

Physicians (particularly GPs) also have a responsibility to educate people. Regarding the problem of health literacy and lack of knowledge of the healthcare system, it may involve providing education and information about the health system and preventive care, improving access to information through language-appropriate materials and resources, and working to increase awareness of the benefits of preventive care. This can help immigrants take an active role in their own healthcare and improve overall health [41, 42].

All these aspects combined leads to the presumption that it is not the migration background per se that is responsible for the significantly lower participation in screening examinations, but factors such as health literacy, language and/or cultural barriers or a general lack of knowledge – which could be tested in future studies.

Furthermore, it is imperative to take into account the underreporting of the heterogeneity that results from the diverse backgrounds and places of origin of the migrant population. From their diverse countries of origin, migrants bring unique cultural, socioeconomic, and healthcare-related influences with them. If this diversity is ignored, then our understanding of the full range of ways in which migration-related factors impact healthcare utilization may be restricted. It is crucial to acknowledge that variations may exist among these groups that call for discussion. In addition, future studies could compare migrants from different origins and destinations while accounting for the diversity within these groups and investigate the long-term impacts of preventive measures on the health and well-being of migrants. As an illustration, it might comprise immigrants from nations within Europe (e.g. the former Yugoslavia) who moved throughout Europe and immigrants who moved to Europe from regions outside of Europe, like the Middle East. It would be beneficial to investigate different combinations of the country of migration and the country of origin. Further research with more recent data could improve our understanding even more, since our study used data from 2014, which predates a notable wave of migration.

## Electronic supplementary material

Below is the link to the electronic supplementary material.

Supplementary Material 1

## Data Availability

The data used in this study are third-party data. The anonymized data sets of the DEAS (1996, 2002, 2008, 2011, 2014, 2017, 2020, 2020/2021) are available for secondary analysis. The data has been made available to scientists at universities and research institutes exclusively for scientific purposes. The use of data is subject to written data protection agreements. Microdata of the German Ageing Survey (DEAS) is available free of charge to scientific researchers for non-profitable purposes. The FDZ-DZA provides access and support to scholars interested in using DEAS for their research. However, for reasons of data protection, signing a data distribution contract is required before data can be obtained. Please see for further information (data distribution contract): https://www.dza.de/en/research/fdz/access-to-data. DEAS-Support is provided by Dr. Stefan Stuth (stefan.stuth@dza.de). Please see for further details: https://www.dza.de/en/research/fdz/contact-and-support.
